# Ethnomedicinal Uses, Phytochemistry, and Therapeutic Potentials of *Litsea glutinosa* (Lour.) C. B. Robinson: A Literature-Based Review

**DOI:** 10.3390/ph16010003

**Published:** 2022-12-20

**Authors:** Sarmin Jamaddar, António Raposo, Chandan Sarkar, Uttam Kumar Roy, Isaac Moura Araújo, Henrique Douglas Melo Coutinho, Ali Saleh Alkhoshaiban, Hmidan A. Alturki, Ariana Saraiva, Conrado Carrascosa, Muhammad Torequl Islam

**Affiliations:** 1 Department of Pharmacy, Bangabandhu Sheikh Mujibur Rahman Science and Technology University, Gopalganj 8100, Bangladesh; 2 CBIOS (Research Center for Biosciences and Health Technologies), Universidade Lusófona de Humanidades e Tecnologias, Campo Grande 376, 1749-024 Lisboa, Portugal; 3 Department of Biological Chemistry, Laboratory of Microbiology and Molecular Biology, Program of Post-Graduation in Molecular Bioprospection, Regional University of Cariri, Crato 63105-000, CE, Brazil; 4 Academic and Training Affairs, Qassim University Medical City, Qassim University, Buraydah 52571, Saudi Arabia; 5 General Directorate for Funds & Grants. King Abdulaziz City for Science & Technology, Riyadh 11442, Saudi Arabia; 6 Department of Animal Pathology and Production, Bromatology and Food Technology, Faculty of Veterinary, Universidad de Las Palmas de Gran Canaria, Trasmontaña s/n, 35413 Arucas, Spain

**Keywords:** *Litsea glutinosa*, ethnobotanical uses, phytochemistry, biological activities

## Abstract

*Litsea glutinosa* (Lour.) C. B. Robinson, belonging to the family Lauraceae, is a multipurpose and fast-growing evergreen or deciduous tree that has been traditionally used for numerous purposes such as treatment for diarrhea, dysentery, abdominal pain, indigestion, gastroenteritis, edema, traumatic injuries, colds, arthritis, asthma, diabetes, pain relief, and poignant sexual power. This study aimed to summarize the chemical reports, folk values, and phytopharmacological activities of *L. glutinosa*, based on available information screened from diverse databases. An up-to-date electronic-based search was accomplished to obtain detailed information, with the help of several databases such as Google Scholar, Scopus, SpringerLink, Web of Science, ScienceDirect, ResearchGate, PubMed, ChemSpider, Elsevier, BioMed Central, and the USPTO, CIPO, INPI, Google Patents, and Espacenet, using relevant keywords. Outcomes advocate that, up to the present time, alkaloids, glycosides, and terpenoids are abundant in, and the most bioactive constituents of, this natural plant. Results demonstrated that *L. glutinosa* has various remarkable biological activities, including antioxidant, anti-inflammatory, anti-microbial, anticancer, antipyretic, anti-diabetic, analgesic, hepatoprotective, and wound-healing activity. One study revealed that *L. glutinosa* exhibited significant aphrodisiac and anti-infertility activity. Nevertheless, no clinical studies have been cited. Taken together, *L. glutinosa* may be one of the significant sources of bioactive constituents that could potentially lead to different effective pharmacological activities. On the other hand, future research should focus on clinical studies and several toxicity evaluations, such as sub-chronic toxicity, teratogenicity, and genotoxicity.

## 1. Introduction

In technologically advanced as well as emerging nations, the practice of using traditional medicine has existed for decades, as a root for the handling of many diseases. Undoubtedly, their significance has been widely known, due to an acquaintance with medicinal plants which includes their indications following native principles and the fact that they are beneficial both for the preservation of cultural backgrounds and biodiversity and for the communal health system and medicinal development now and in the future [1,2]. To treat infections, medicinal plants and their extracts have been used since ancient times and have also become an imperative part of the title role in newer therapeutic agents’ discovery [3,4]. Due to the cost effectiveness and ecofriendly characteristics, there is growing interest in the use of herbal medicine in pharmaceutical consumption [5]. Traditional medicine contains a wide class of phytochemicals that exert several biologic activities, including antibacterial, antidiabetic, antifertility, antifungal, anti-hypercholesteremic, anti-inflammatory, antitumor, cardiovascular, central nervous-system depressant, cytotoxicity, diuretic, and others that are dedicated to treating diverse human diseases [6].

*Litsea glutinosa* (Lour.) C.B. Robinson, belongs to the family Lauraceae, and is a medicinal plant of immense pharmaceutical value that reaches a height of approximately 3–15 m and whose bark is 2–2.5 cm thick, with a brown surface [7]. It is a polymorphic species with leaves that are simple, elliptical-to-oblong-elliptical, pilose when young, 3.5–10 × 1.5–11 cm, and with unisexual flowers that are yellow in color and 5–6 mm across, along with fruits, a berry that is 5–6 mm across and which may be purple, on a flat disc [8,9]. *L. glutinosa* is native to India, South China, Malaysia, Australia, the western Pacific Islands, Bhutan, Myanmar, Nepal, Philippines, Thailand and Vietnam, in the forest of Chittagong and the Sylhet districts in Bangladesh [7,10]. *L. glutinosa* is known as Maida Lakri, sycamore, Indian laurel [English]; bolly beech, brown bollygum, brown bollywood, brown beech, soft bollygum [English/Australia]; Indiese lourier [Afrikaans]; avocat marron, litsée glutineuse [French]; bois d’oiseau [French/Mauritius]; puso-puso, sablot [Tagalog]; bời lời đỏ [Vietnamese]; কুকুরচিতা [Bengali]; 潺槁木姜子 [Chinese]; หมูทะลวง [Thai] [7,8,11,12].

Traditionally, the bark acts as one of the most prevalent folk medicines, exerting medicinal values for treating diarrhea, dysentery, abdominal pain, indigestion, gastroenteritis, edema, traumatic injuries, colds, arthritis, asthma, diabetes and as a treatment for pain relief and for poignant sexual power [8,13], while the leaves exert antibacterial and cardiovascular activities, as well as the extreme flow of semen in male [7]. The leaves can also be used as a topical medicine to heal wounds and bruises, as well as providing an emollient action to relieve the stresses of rheumatic and gouty joints [11]. *L. glutinosa* contains a variety of essential oils that act as antibacterial agents [13]. The phytochemical investigation of *L. glutinosa* bark proves the presence of a variety of important phytochemical compounds, including alkaloids, glycosides, flavonoids, diterpenes, phenols, amino acids, carbohydrates, proteins, and saponins in hydroalcoholic extracts [13].

This study aims to carry out a phytochemical investigation as well as pharmacological studies of *L. glutinosa* as a new therapeutic medicine.

PLANT TAXONOMY

The taxonomical classification of *L. glutinosa* is the following:

Domain: Eukaryota

Kingdom: Plantae

Phylum: Spermatophyta

Subphylum: Angiospermae

Class: Dicotyledonae

Order: Laurales

Family: Lauraceae

Genus: *Litsea*

Species: *Litsea glutinosa*

PLANT MORPHOLOGY

*Litsea glutinosa* is a small to a medium-sized plant which can be 3–20 m tall with straight or curved stems up to 60 cm in diameter. According to Mohammad et al. (2020) this plant species contains a mean tree-height of 13.24 (m), mean clear-bole height of 4.61 (m), mean girth at breast height of 62.89 (cm), mean crown-radius of 2.51 (m), mean crown-diameter of 5.17 (m), mean crown-height of 8.63 (m), mean number of primary branches, 5.46, mean leaf area, 91.09 (cm^2^), mean leaf-weight, 0.55 (gm), mean specific leaf-area, 175.7 (cm^2^/g), and mean bark wt./unit area, 2.75 (g) [8]. This species contains gray-yellow silky young branchlets, on which leaves are oval-shaped but adjustable and of 7–15 × 3–7 cm, alternately arranged and 1–2.6 cm long [10]. The leaf centers are wedge-shaped, and blunt or curved, while, on the contrary, the fruits are round in shape and of approximately 5–7 mm; 85% of the germination of seeds occurs in 15–45 days, and flowers bloom from May to June [7]. Male flowers can be recognized by their imperfect or missing petals and a productive stamen contains not less than 15 flowers [7].

## 2. Results

### 2.1. Traditional and Folk Values

The folk value of a medicinal plant is very important, because traditional use leads to the development of a new therapeutic drug. Most of the population of rural areas are dependent on medicinal plants for the treatment of various diseases [14]. Although all the parts of *L. glutinosa* have been used to treat a diversity of ailments, the bark has played the most efficient role over almost all its distribution range. The bark has been used as a binding agent in tablet formulation, as plasters for fractured limbs, for relieving pain, for a soothing effect on the body in case of skin infection, for arousing sexual power and acting as an aphrodisiac, as well as healing wounds on the neck of bullocks and stopping bleeding. It is also conventionally used as an energy tonic by some individuals [15]. Traditionally, a paste of its bark powder and the mucilage in the gum from the bark is used to treat or comfort these problems [16].

The leaf powder of this plant has been used to treat stomach problems such as diarrhea and dysentery, as well as to heal wounds, bruises, swelling, furunculosis, reduce fever, and so on. The mucilage of the leaves is applied to cleanse hair and scalp [15]. The leaves of this plant also act as an antispasmodic, as well as an emollient [17,18]. The extraction of essential oil from the berries of this plant is used to treat rheumatism [17,18] and the leaf essential-oil acts as an antiseptic agent [19]. The seed powder is used to treat skin boils, and, on the other hand, the root paste has the ability to poultice sprains and bruises, heal fever, swelling, and furunculosis [20]. Table 1 summarizes traditional uses of *L. glutinosa*.

### 2.2. Phytochemistry

**Figure 1:**
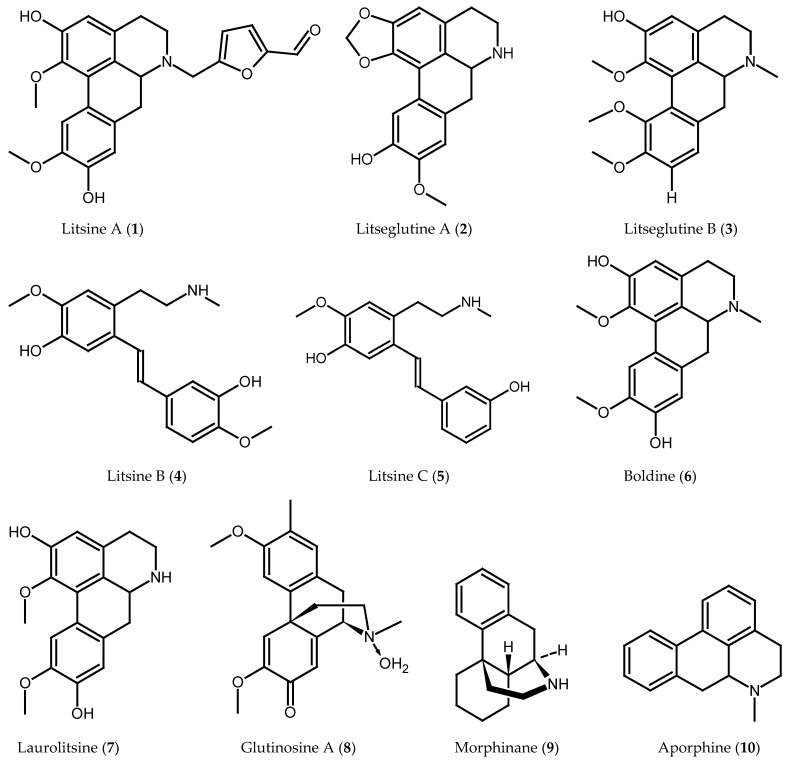

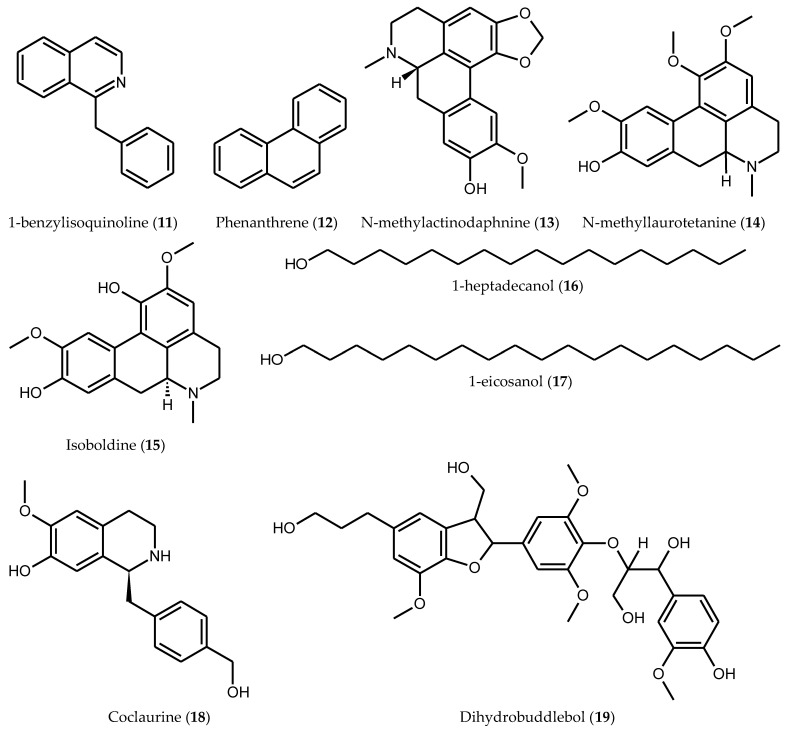

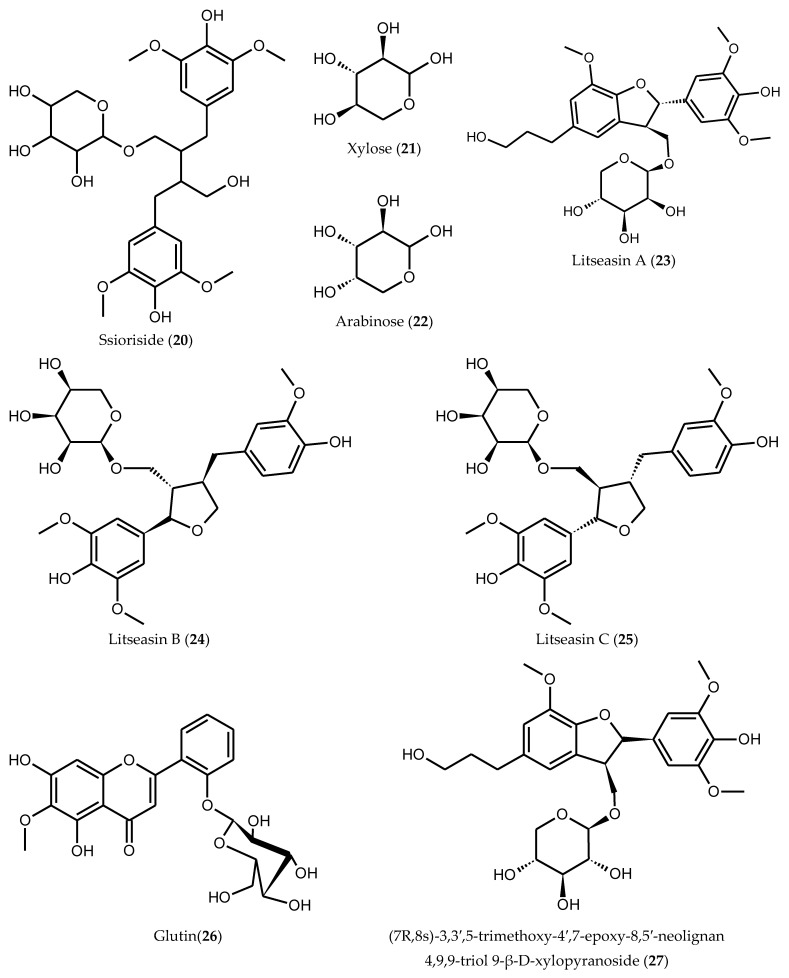

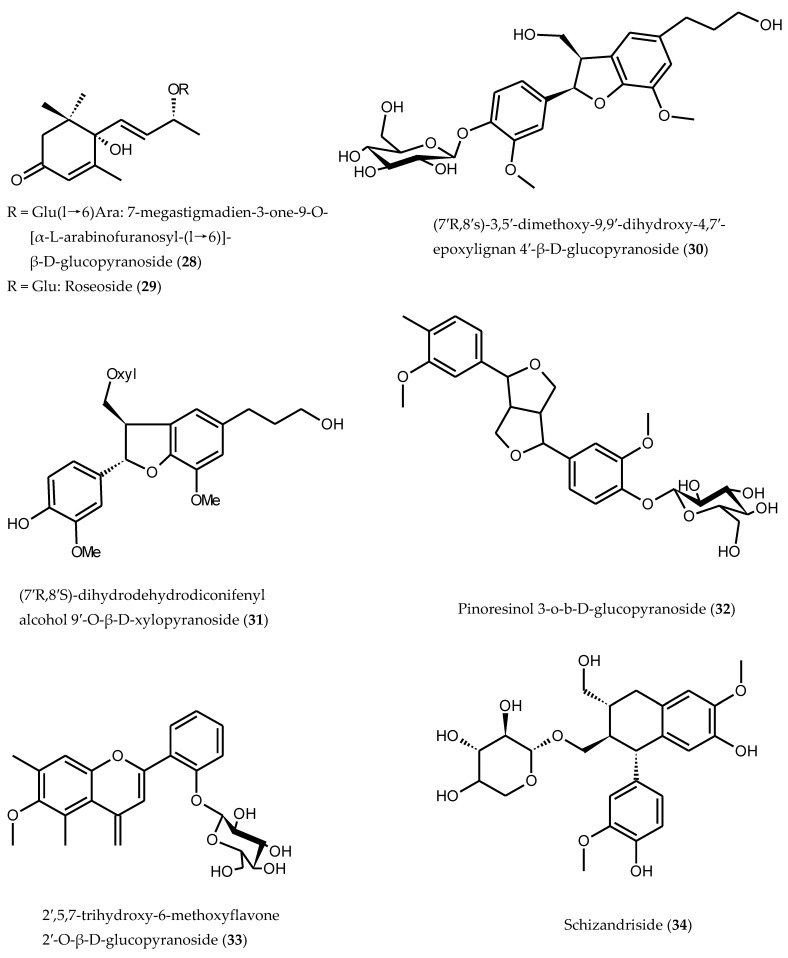

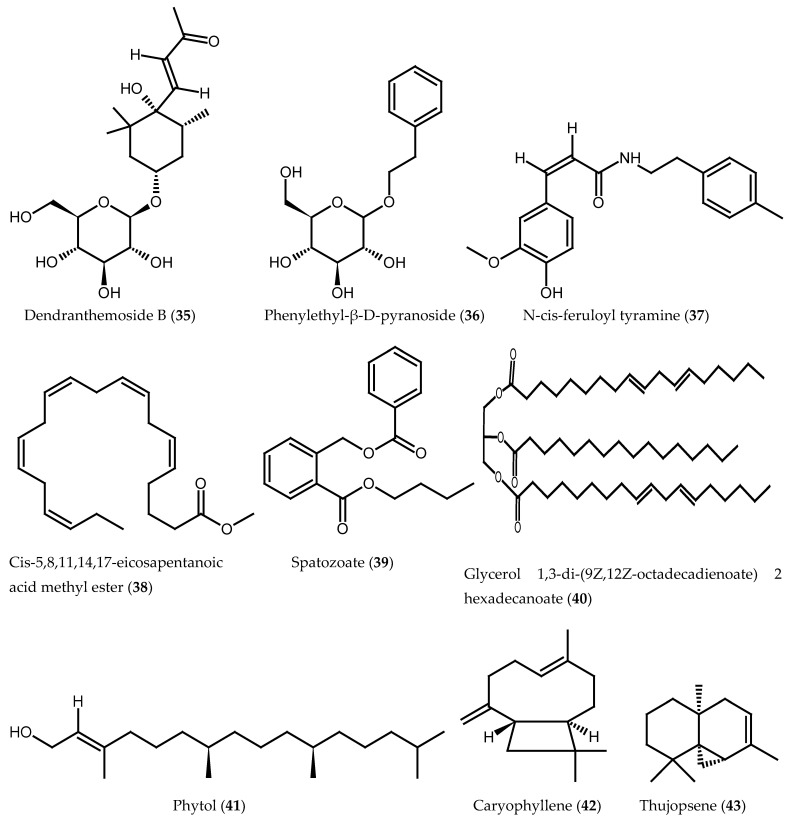

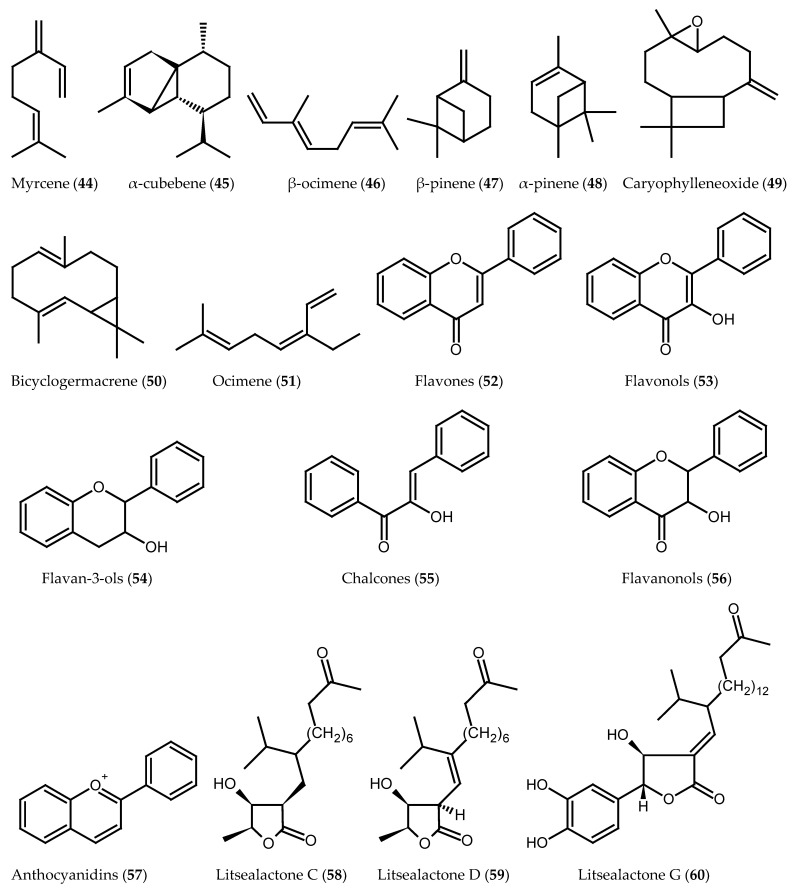

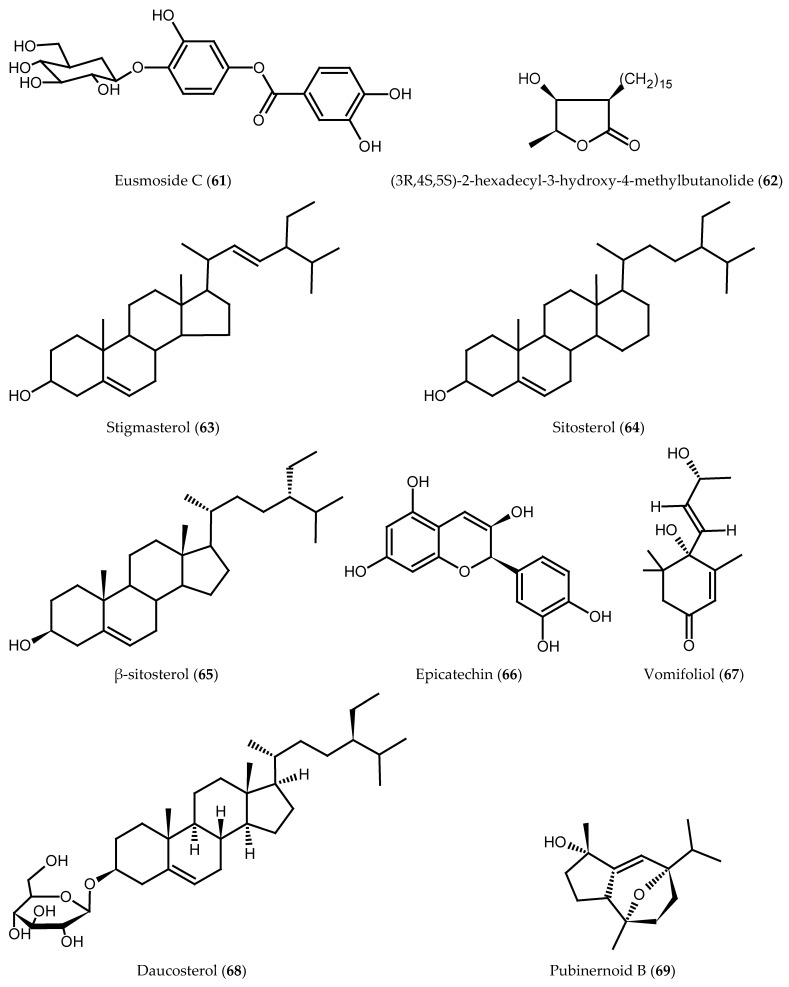

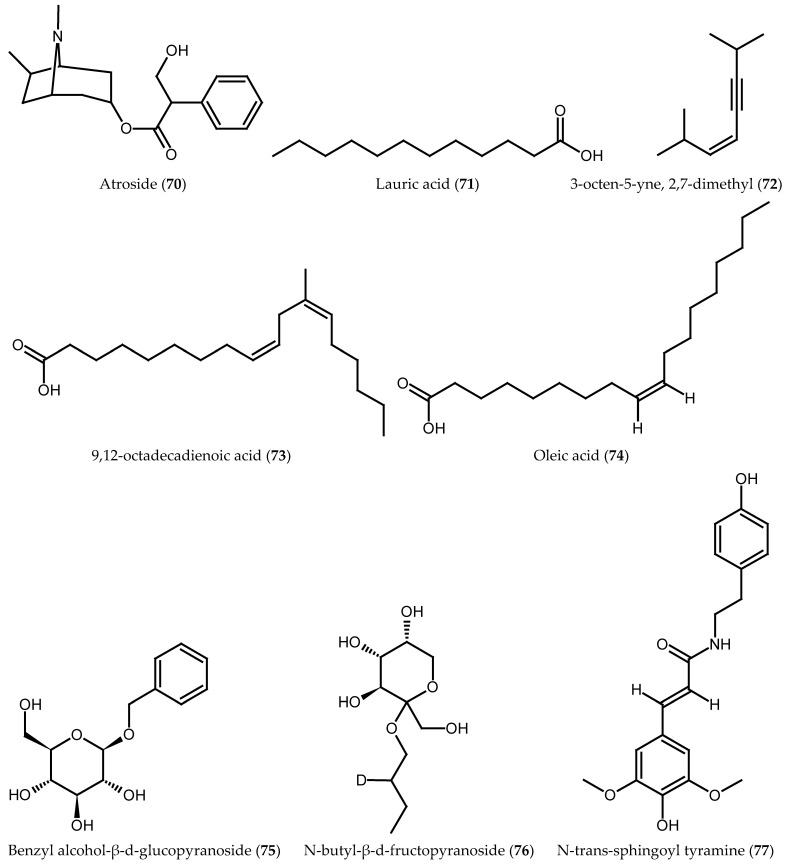
Chemical structure of some important isolated compounds from *Litsea glutinosa*.

Due to the presence of some organic compounds in this medicinal plant, it can provide certain biological actions on the human body, most of which seem to be non-essential for growing the plant itself [23]. These organic substances, or phytochemical constituents, can be alkaloids, alcohols, carbohydrates, glycosides, esters, essential oils, flavonoids, lignans, lactones, steroids, tannins, terpenoids, and so on. Their structures and resources have been comprehensively summarized and are represented in Figure 1 and Table 2. Based on these results, we can conclude that alkaloids, glycosides, and terpenoids are abundant in and bioactive constituents of this natural plant.

#### 2.2.1. Alkaloids

Recently, research on the bio-potential of heterocycles containing nitrogen has been updated [35]. Alkaloids are compounds found in plants that include one or more nitrogen atoms and are typically found in cyclic systems. Similarly, this is true for these nitrogen-containing heterocycles, which include isoquinoline alkaloids and their N-oxides, as sources of drug-discovery leads [36]. *L. glutinosa* is also found as a source of these types of compounds such as litsine A (**1**), litseglutine A (**2**), litseglutine B (**3**), litsine B (**4**), litsine C (**5**), boldine (**6**), laurolitsine (**7**), glutinosine A (**8**), morphinane (**9**), aporphine (**10**), 1-benzylisoquinoline (**11**), phenanthrene (**12**), N-methylactinodaphnine (**13**), N-methyllaurotetanine (**14**), and isoboldine (**15**) [13,24,25,26,27,28,29,30]. Among all presented aporphine alkaloids in this plant such as litsine A-C (**1,4,5**) and litseglutine A-B (**1,2**), litsine A (**1**) exhibits potent activity in increasing glucose-uptake [24,25], whereas glutinosine A (**8**), morphinandienone alkaloid isolated from the root bark, shows no activity in stimulating glucose-consumption [27]. Moreover, N-methylactinodaphnine (**13**) is the most cytotoxic among four known aporphine alkaloids including N-methylactinodaphnine (**13**), boldine (**6**), N-methyllaurotetanine (**14**) and isoboldine (**15**), and this observation may be explained by the presence of a 1,2-methylenedioxy group [28]. A new aporphine-type alkaloid, proaporphine, is very common in this plant, and has a significant role as an antibacterial and antifungal agent [37]. On the other hand, the phenanthrene-type alkaloid morphine obtained from the root bark of this plant is frequently regarded as a model opioid analgesic and the standard by which all other analgesics are measured [38].

#### 2.2.2. Alcohols

Lin et al. have identified five alcohols from the natural plant *L. glutinosa* , including 1-heptadecanol (**16**), 1-eicosanol (**17**), Coclaurine (**18**), Dihydrobuddlenol (**19**), and Ssioriside (**20**) [29,30].

#### 2.2.3. Carbohydrates

Only two carbohydrates, (xylose (**21**) and arabinose (**22**)) have been isolated from the green leaves of *L. glutinosa* [31]. It was discovered that the molar ratio of xylose and arabinose in a water-soluble novel arabinoxylan, which was isolated using hot water extraction from the green leaves of *L. glutinosa*, was approximately 1:3 [31].

#### 2.2.4. Glycosides

Approximately 13 glycosides have been reported from the root, leaf, and twig parts of *L. glutinosa*. Wu et al. (2017) investigated the chemical constituents in its root barks for the first time. Three new lignan glycosides named Litseasins A-C (**23–25**), together with the known one, (7R,8S)-3,3′,5-trimethoxy-4′,7-epoxy-8,5′-neolignan4,9,9-triol 9-β-D-xylopyranoside (**27**), were isolated [32]. Phytochemical study on the leaves and twigs afforded the new megastigmane diglycoside (6S, 7E, 9R)-6, 9-dihydroxy-4, 7-megastigmadien-3-one-9-O-[α-L-arabinofuranosyl-(l→6)]-β-D-glucopyranoside (**28**), along with roseoside (**29**), (7′R, 8′R)-3, 5′-dimethoxy-9, 9′-dihydroxy-4, 7′-epoxylignan 4′-β-D-glucopyranoside (**30**), (7′R, 8′S)-dihydrodehydrodiconifenyl alcohol 9′-O-β-D-xylopyranoside (**31**) and pinoresinol 3-O-β-D-glucopyranoside (**32**) [39]. Among these, (6S, 7E, 9R)-6, 9-dihydroxy-4, 7-megastigmadien-3-one-9-O-[α-L-arabinofuranosyl-(l→6)]-β-D-glucopyranoside (**28**) was evaluated for cytotoxic activities against human tumor cell-lines (myeloid leukemia HL-60, hepatocellular carcinoma SMMC-7721, lung cancer A-549, breast cancer MCF-7 and colon cancer SW480 cells) [39]. A total of four steroids have been identified exclusively in the root bark of *L. glutinosa*, with schizandriside (**34**), dendranthemoside B (**35**), phenylethyl-β-D-glucopyranoside (**36**), and N-cis-feruloyl tyramine (**37**) being the most representative compounds [30].

#### 2.2.5. Esters

From the ethyl acetate extract of the barks of *L. glutinosa*, three ester compounds, including *cis*-5,8,11,14,17-eicosapentaenoic acid methyl ester (**38**), spatozoate (**39**), and glycerol 1,3-di-(9Z,12Z-octadecadienoate) 2-hexadecanoate (**40**) have been isolated [29].

#### 2.2.6. Terpenes

A modified version of terpenes known as “terpenoids” may be used to create a “flavor fingerprint” of plant species that is typically recognized by animals and humans [40]. Turpentine is the source of the terms “terpenes” and “terpenoids”. The C-10 terpenoids (Monoterpenes) or the five-carbon-units interpenoids, also known as isoprene units (which give off the gas isoprene at high temperatures), were formerly assumed to be the smallest group of this class [41]. Terpenes are the components of vital oil, and have significant biological functions. In light of this, around 16 terpenes were exported from the leaves, barks, and fruits of *L. glutinosa* [17]. Monoterpenes mainly occur in volatile oil, and are identified using GC-based techniques. Many papers have described the GC analysis of volatile oil from different parts of *L. glutinosa*, including myrcene (**44**), α-cubebene (**45**) β-ocimene (**46**), β-pinene (**47**), α-pinene (**48**), and ocimene (**51**) [17]. Researchers have been interested in nine sesquiterpenes [17] extracted from different parts of *L. glutinosa*, due to their wide range of biological activities, as demonstrated by their anti-oxidative, anti-fungal, anti-asthmatic, anti-anaphylactic, and central-nervous-system (CNS) functions [13]. In addition, one diterpenoid, exemplified by phytol (**41**), has been isolated from *L. glutinosa* [17].

#### 2.2.7. Flavonoids

The commonly utilized species of Litsea are significant producers of flavonoids. The plant of *L. glutinosa* is one of the primary sources of flavonoids, which are categorized as flavones (**52**), flavonols (**53**), flavan-3-ols (**54**), chalcones (**55**), flavanonols (**56**), and anthocyanidins (**57**) [13]. Most flavones (**52**), and flavonols (**53**) are found as glycosides, which are composed of glucose, galactose, and rhamnose.

#### 2.2.8. Lactones

Litsealactone C (**58**), Litsealactone D (**59**), Litsealactone G (**60**), (3R,4S,5S)-2-hexadecyl-3-hydroxy-4-methylbutanolide (**62**), and a novel benzoic-acid derivative termed eusmoside C (**61**) were all isolated and characterized as a result of phytochemical studies of a methanolic extract taken from the heartwood of the *L. glutinosa* [11].

#### 2.2.9. Steroids

The biological world, including the kingdom of plants, is abundant in cyclopentane perhydrophenanthrene derivatives, which make up a sizable class known as steroids. Eight steroid chemicals, of which stigmasterol (**63**), sitosterol (**64**), β-sitosterol (**65**), epicatechin (**66**), vomifoliol (**67**), daucosterol (**68**), pubinernoid B (**69**), and atroside (**70**) are the most typical, have been found only in the bark and aerial portions of *L. glutinosa* [15,29,33,42].

#### 2.2.10. Miscellaneous Constituents

Many other constituents have been obtained, such as lauric acid (**71**), 3-octen-5-yne, 2,7-dimethyl (**72**), 9,12-octadecadienoic acid (**73**), oleic acid (**74**), benzyl alcohol-β-d-glucopyranoside (**75**), N-butyl-β-d-fructopyranoside (**76**), and N-trans-sphingoyl tyramine (**77**) [17,30].

### 2.3. Pharmacological Properties

Although there is a wealth of medicinal information on the plant *L. glutinosa* in the literature, many pharmaceutical activities simply lack relevant comparisons with positive controls. *L. glutinosa* has been studied for a long time for its ethnopharmacological potential, due to its widespread therapeutic applications in traditional systems, and several reviews have incorporated this information. The investigations conducted were also appropriate in terms of the source of materials employed, the test system, the minimal effective-dosage, the relevant pharmacological-doses of active extracts, and the possible mechanisms that are listed in Table 3.

#### 2.3.1. Antioxidant Activity

According to certain research, *L. glutinosa* extracts exhibit antioxidant- properties and can modify oxidative stress, which may be helpful for treating a variety of conditions connected to oxidative stress, including diabetes, cancer, and wound healing [52]. Numerous studies have shown that *L. glutinosa* and its derivatives have strong antioxidant properties both in vitro and in vivo, because they include a variety of bioactive elements, such as polyphenols and peptides.

A study reported that the antioxidant capability of the methanolic extract increases in a concentration-dependent manner in the range of 50–250 mg/mL [43]. In another study, *L. glutinosa* leaf-extract showed a concentration-dependent DPPH free radical scavenging capacity [7]. In this study, the authors also determined total phenolic-content and reducing-power capacity of the extract.

#### 2.3.2. Anti-Inflammatory Activity

Inflammation is the body’s reaction to tissue damage or its defense against infectious diseases or other threats. A steady change in the kind of cells present at the inflammatory site, on the other hand, characterizes chronic inflammation, a long-term inflammatory response. This can happen after an acute or low-grade form of inflammation, and is defined by the simultaneous destruction and remodeling of tissue as a result of the inflammatory process [53]. Additionally, inflammation has a role in the pathophysiology of numerous illnesses, such as diabetes, cancer, and liver disorders [54].

Using the rat-paw edema model, the methanolic extract of *L. glutinosa* leaves at doses of 250 and 500 mg/kg showed protection against carrageenan-induced paw edema by inhibiting either cyclooxygenase and/or lypooxygenase enzymes [7].

#### 2.3.3. Anti-Microbial Activity

Due to the inappropriate use of antibiotics, a number of pathogenic microbes have evolved different antibiotic resistances, and the threat of antimicrobial resistance is expanding at an alarming rate. As a result, antibiotic resistance has emerged as a significant global health issue. In this situation, researchers are looking for novel therapeutic drugs to combat harmful germs. A viable source might be medicinal plants, because of their powerful pharmacological effects, cost-effectiveness, and lack of negative side-effects [55].

Using the disc-diffusion assay method, the ethanolic extract of *L. glutinosa* leaves was assessed for its antibacterial effect in vitro against pathogens that cause urinary tract infections, such as *Staphylococcus aureus*, *Pseudomonas aeruginosa*, *Proteus mirabilis*, *Enterococcus faecalis*, and *Escherichia coli*. With zones of inhibition ranging from 8.1 to 11. 8 mm, the extract at a concentration of 250 g/disc demonstrated excellent inhibition against these tested pathogens [19]. Methanol extract from the bark of *L. glutinosa* was tested for its antibacterial properties using the agar-diffusion technique, by Mandal et al. (2000). Both gram-positive and gram-negative bacteria were inhibited by this extract, and the zones of inhibition ranged from 6.5 to 13.5 mm, which was equivalent to the positive control chloramphenicol [44]. The antibacterial activity of the stem bark and leaf extracts of *L. glutinosa* was also evaluated against *S. aureus*, *Bacillus stubtilis*, *E. coli*, *Pseudomonas aeruginosa*, *Klebsiella pneumoniae*, *Staphylococcus typhi*, *Salmonella paratyphi* and *Proteus* sp. with the help of the agar-well diffusion method [45]. Haque et al. reported that ethanolic leaf-extract (1000 μg/disc) was found to show better anti-bacterial activity against *E. coli* (zone of inhibition of 30 mm) than the distilled-water extract [15].

In another study, the results indicated that extracts (hexane, chloroform, and methanol) of *L. glutinosa* possess good antimicrobial activity with significant minimum-inhibitory-concentration (MIC) values against *E. faecalis*, *P. aeruginosa*, and *Staphylococcus pneumoniae* at 31.2 μg/mL [8]. In addition, methanolic leaf-extract and ethanolic bark-extract [46] of *L. glutinosa* showed effective antimicrobial effects in a dose-dependent manner against the test microbes. Moreover, the leaf extract was also tested for larvicidal activity on 3rd instar *Aedes aegypti* larvae, and a lethal concentration 50 (LC_50_) was assessed at 15.43 g/L [47]. This research suggested that this extract could be used as a potential biological-control agent against *A. aegypti* mosquito larvae.

#### 2.3.4. Antipyretic Activity

There were 14.1 million new cases of cancer, 8.2 million deaths from cancer, and 32.6 million people living with cancer (within 5 years of diagnosis) in 2012, according to estimates by the International Agency for Research on Cancer of the incidence of mortality and prevalence of major types of cancer at the national level for 184 countries around the world [56]. There will be 26 million new instances of cancer and 17 million annual cancer deaths by the year 2030, according to estimates [57]. Therefore, there is a continuing need to provide novel, efficient, and cost-effective anticancer medications [58]. Medicinal plants and their chemical constituents have been utilized to treat human ailments since the beginning of ancient medicine.

A new megastigmane diglycoside (6S, 7E, 9R)-6, 9-dihydroxy-4, 7-megastigmadien-3-one-9-O-[α-L-arabinofuranosyl-(l→6)]-β-D-glucopyranoside isolated from leaves and twigs of *L. glutinosa* was evaluated for cytotoxic activities against human tumor cell-lines (myeloid leukemia HL-60, hepatocellular carcinoma SMMC-7721, lung cancer A-549, breast cancer MCF-7 and colon cancer SW480 cells), for which it was proved to be inactive (IC_50_ > 40 µM) [39]. The most cytotoxic aporphine-alkaloid, N-methylactinodaphnine, was discovered from this plant, and this finding may be explained by the presence of a 1,2-methylenedioxy group. A conceivable explanation for the observed cytotoxicity, according to in silico docking, is the stabilization of a topoisomerase II (β) DNA-enzyme complex [28]. In in vitro studies on Saos-2 cells, methanolic extract of *L. glutinosa* bark significantly downregulates the apoptotic and proliferative markers in Saos-2 osteocytes [48].

#### 2.3.5. Anti-Pyretic effect

The term “pyrexia” or “fever” refers to an elevation of body temperature above the normal physiological range. Pyrexia or fever can be caused by a variety of physiological stressors, including ovulation, increased thyroid secretion, excessive exercise, any lesions to the central nervous system, leukemia, and most common microbial infections. These days, people favor medicinal plants to treat fever, as they contain natural products that are effective, chemically balanced, and have fewer side effects as compared to synthetic chemicals.

It has been cited that the n-hexane, ethyl acetate, chloroform, and crude methanolic extracts of *L. glutinosa* leaves (500 mg/kg dose) exerted a notable reduction in yeast and provoked an elevation of body temperature (32.78 ± 0.46 °C) through the inhibition of prostaglandin synthetase within the hypothalamus [7].

#### 2.3.6. Anti-Diabetic Effect

The most prevalent endocrine illness, diabetes mellitus (DM), affects more than 100 million individuals globally (6% of the population), and in the next ten years its prevalence may increase by roughly five times [59]. Many of the medications that are now on the market have been either directly or indirectly produced from plants, which have historically been an excellent source of pharmaceuticals. Approximately 800 plants are included in the ethnobotanical database as having possible anti-diabetic properties [59].

Zhang et al. (2018) investigated the fact that an orally administered (50, 100, and 200 mg/kg) alkaloid-rich extract from *L. glutinosa* barks to ob/ob mice for 4 weeks possessed potential anti-hyperglycemic and anti-hyperlipidemic effects, and could be utilized as an effective agent for the treatment of type 2 diabetes [49]. In another study, glutinosine A (10 μM) isolated from the *L. glutinosa* root barks did not stimulate glucose consumption capacity of HepG2 cells [27]. Laurolitsine, an aporphine alkaloid from *L. glutinosa*, was found to have potent antihyperglycemic and antihyperlipidemic effects in ob/ob mice at a high concentration, in the gastrointestinal tract, liver, lungs, and kidneys (26 015.33, 905.12, 442.32, and 214.99 ng/g at 0.5 h, respectively), and low excretion of parent laurolitsine in urine and feces (0.03 and 1.20% at 36 h, respectively) [50]. In addition, a new aminoethylstilbene isoquinoline alkaloid denoted litsine C, isolated from an ethanol extract from the root bark of *L. glutinosa*, was tested for its effect on glucose consumption in HepG2 cells at different concentrations (1–20 μM), and found to significantly increase the glucose uptake [26]. Furthermore, litsine A isolated from the root barks of *L. glutinosa* increased glucose uptake at 10 μM on the glucose-uptake assay on C2C12 myoblasts [24].

#### 2.3.7. Analgesic Activity

A vast number of medicinal plants are thought to provide a wide range of pharmacological effects because they contain a variety of phytochemicals. It is believed that current analgesics, such as opiates and non-steroidal anti-inflammatory drugs, are not always beneficial, because of their adverse effects and limited effectiveness [60].

The n-hexane, ethyl acetate, chloroform, and crude methanolic extracts of *L. glutinosa* leaves (250 and 500 mg/kg) displayed significant analgesic activity in the acetic acid-induced-writhing and hot-plate tests in mice [7]. It has been reported that leaf extracts of *L. glutinosa* at different concentrations (100, 200, and 300 mg/kg) provided significant analgesic activity by inhibiting prostaglandin synthetase, specifically endoperoxidase, using abdominal-writhing and tail-flick methods [43,45].

#### 2.3.8. Hepatoprotective Effect

Several toxic compounds, including chemotherapeutic drugs, thioacetamide, carbon tetrachloride (CCl_4_), certain antibiotics, excessive alcohol use, and pathogenic microorganisms, can cause liver disorders, which have grown into a significant worldwide health problem. Despite developments in pharmacology, the drawbacks of synthetic medications have outweighed their benefits. Thus, research into alternative therapeutic agents for illnesses that do not require excessive cost and time-consuming pharmaceutical-production procedures appears to have attracted international interest.

The hepatoprotective activity of oral administration of the methanol extract of *L. glutinosa* (100–200 mg/kg) was investigated against paracetamol and CCl_4_, which were comparable with silymarin, which was used as a reference standard. The results of this study indicated that this extract offers a significant dose-dependent protection of liver damage against paracetamol- and CCl_4_-induced hepatic damage in rats [51].

#### 2.3.9. Miscellaneous Effects

*L. glutinosa* bark-extract exhibited significant aphrodisiac and anti-infertility activity against immobilization-stress-induced male Wistar albino rats [46]. Bhowmick and his colleagues reported that *L. glutinosa* has a significant ability to disrupt blood clots [7].

### 2.4. Toxicological Profile

Up to a concentration of between 5 and 320 g/mL, the examined cell lines are not significantly cytotoxic when treated with the stem-bark ethanol extract of *L. glutinosa*. Lethality was not seen in the acute-toxicity investigation up to 3000 mg/kg b.w. between the control and treated groups. No discernible variations in body and organ weights or histopathological analyses were found [61].

## 3. Materials and Methods

An electronic-based search was carried out to obtain the following information about the literature of *L. glutinosa* using the databases Google Scholar, Scopus, SpringerLink, Web of Science, ScienceDirect, ResearchGate, PubMed, ChemSpider, Elsevier, BioMed Central, USPTO, CIPO, INPI, Google Patents, and Espacenet. The key words ‘*Litsea glutinosa*’ were paired with ‘phytochemicals’, ‘traditional use’, ‘folk values’, ‘morphology’, ‘fruits’, ‘leaves’, ‘bark’, ‘medicinal use’, ‘pharmacology’, ‘toxicology’, ‘crude extracts’, ‘herbal drugs’, etc., to obtain published-literature archives. Verbal constraints were not mandatory. The data obtained in this study were included or excluded, in accordance with the following criteria.

Data-inclusion criteria included: (a) studies related to in vitro, ex vivo or in vivo with or without using experimental animals as well as humans and their isolated cells and tissue; (b) studies related to the pharmacology and phytochemicals derived from this plant; (c) toxicological-profile evaluation along with the morphology of this plant were also included; (d) single or multiple cell-lines or animals used in the study; (e) proposed mechanism of this study; (f) *L. glutinosa* in other studies dealing with the up-to-date topic.

**Figure 2:**
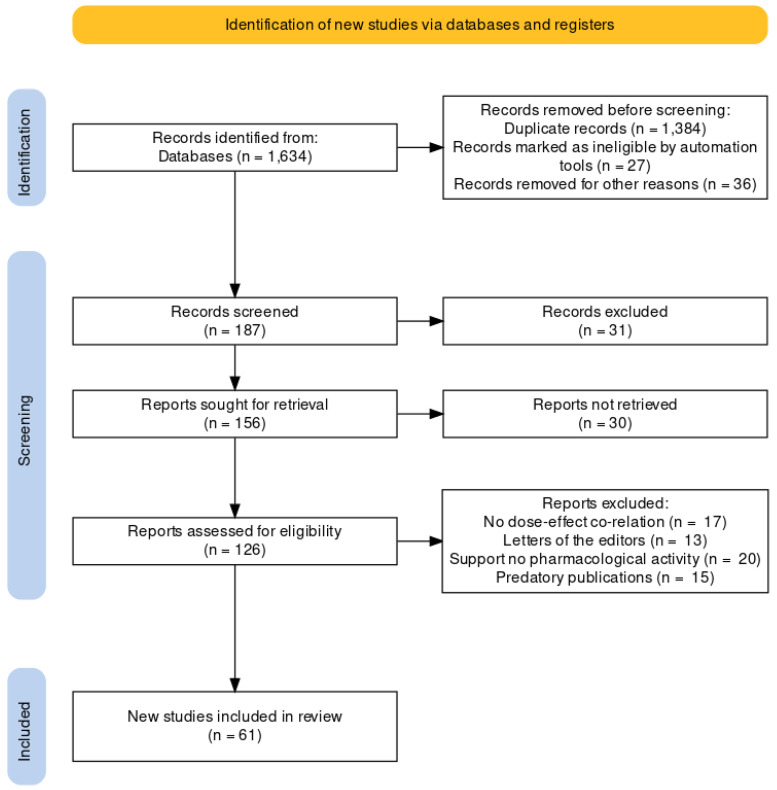
PRISMA flow diagram.

Exclusion criteria: (a) repetition of data and titles and/or summaries that do not meet the inclusion criteria; (b) reports on other related plants from the same species Litsea, similar to *L. glutinosa* extract. After refining the study through the inclusion and exclusion criteria, 61 articles were chosen. This study deals with the pharmacology, traditional values, and phytochemicals, as well as discovering further research on this plant. Figure 2 indicates the flow diagram of this study.

## 4. Conclusions and Future Perspectives

One of the oldest plants in the world, *L. glutinosa*, has captivated people’s attention for centuries, thanks to its incredibly promising potential as a nutritious food and medicine. Recent phytochemical studies, particularly those conducted in the last two decades, have extracted many significant bioactive compounds from the leaves, stem bark, fruits, and roots of *L. glutinosa*, which have been shown to contribute potent pharmacological activities. The known scientific publications on the phytochemistry and pharmacological properties of *L. glutinosa*, as well as its ethnomedicinal usage, were all discussed in the current review. This study mainly focused on the traditional and folk values, phytochemical constituents, and pharmacological properties of *L. glutinosa*. *L. glutinosa* exhibited a variety of biological activities, including antioxidant, anti-inflammatory, antimicrobial, anticancer, antipyretic, anti-diabetic, analgesic, hepatoprotective, and so on.

There are some important pharmacologically active lead-compounds of *L. glutinosa,* for example litsine A (1), which exhibits potent activity in increasing glucose uptake, while *N*-methylactinodaphnine (13) and (6S, 7E, 9R)-6, 9-dihydroxy-4, 7-megastigmadien-3-one-9-O-[α-L-arabinofuranosyl-(l→6)]-β-D-glucopyranoside (28), act as potent cytotoxic agents against human tumor cell-lines (myeloid leukemia HL-60, hepatocellular carcinoma SMMC-7721, lung cancer A-549, breast cancer MCF-7, and colon cancer SW480 cells). Moreover, some sesquiterpenes represent anti-oxidative, anti-fungal, and neuropharmacological activities. This review depicts that phytochemicals of *L. glutinosa* have potential sources of phytotherapeutic lead-compounds, thus, this herb might be one of the valuable alternative sources for treating a variety of disorders.

Although some of the ethnomedicinal claims about *L. glutinosa*’s bioactivities may be supported by scientific research, the available data are limited and tentative, to some extent. More crucially, via more in vivo and clinical investigations, a variety of problems and difficulties should be resolved in order to close the scientific knowledge gap for *L. glutinosa*. Firstly, future studies should include common pharmacological approaches and parameters, especially for anticancer, antidiabetic, and hepatoprotective activities, such as positive and/or negative controls, normal cells, dose- and time-dependent relationships, and maximum and minimum dose-response, or time-response, in order to provide crucial pharmacological information about *L. glutinosa* and facilitate the reproducibility of the data. Additionally, the mechanism of action of *L. glutinosa* and its bioactive components is currently understood to be basic, which, to a certain extent, could weakly support its traditional usage (as discussed in the anticancer, antidiabetic, and hepatoprotective sections). The information that is now available, which is restricted to the extracts, is insufficient to evaluate and understand the precise mechanisms underpinning the bioactivities of *L. glutinosa* and its bioactive components. Future research should thus concentrate on and investigate many potential molecular pathways, rather than being limited to just one mechanism, in order to support the numerous ethnomedicinal claims made for *L. glutinosa*.

Future research should examine the structure–function relationships and modes of action of the bioactive elements in connection with their pharmacological activity, such as the antiviral properties against *A. aegypti* mosquito larvae. Researchers are also urged to use sophisticated chromatography and spectroscopy techniques, such as nuclear magnetic resonance (NMR) and liquid chromatography/time-of-flight mass spectrometry (LC/TOF-MS), which are preferred over the traditional thin-layer chromatography (TLC) and high-performance liquid chromatography (HPLC) methodologies. In addition, the majority of phytochemical and pharmacological studies focus on the leaves, stem barks, and roots of *L. glutinosa*; hence, it is recommended that researchers conduct pharmacological studies on other parts of this plant, such as fruits, seeds, and flowers. As the current data are limited to the toxicity of different extracts of this plant, future research should focus on several toxicity-evaluations such as acute toxicity, subacute toxicity, subchronic toxicity, teratogenicity, and genotoxicity, for long-term intake purposes.

It would be advantageous for pre-clinical and clinical studies to assess the pharmacokinetics and toxicokinetics of various extracts and bioactive ingredients on the target organ. Future toxicological research must also take into account the distinct pharmacological activity of the extracts or bioactive ingredients. It is intended that the current analysis will enlighten, and provide the framework and direction for, researchers in crucial areas as they perform more in vitro, in vivo, and clinical examinations of *L. glutinosa* and its future development as an enhanced treatment.

## Figures and Tables

**Table 1:** Traditional uses of *Litsea glutinosa*.

Traditional Uses	Part Used	Mode of Administration	References
Antispasmodic, emollient, poultice, diarrhea, dysentery as well as for wounds and bruises, fever, swelling, furunculosis	Leaves	Leaf powder	[17,18]
Cleaning the hair and scalp	Leaves	Clear mucilage solution	[21]
Rheumatism	Berries oil	Essential oil	[17,18]
Antiseptic	Leaves	Essential oil	[22]
Energy tonic	Bark		[15]
Binding agent in tablet formulations, as plasters for fractured limbs, treating pain, aphrodisiac or to arouse sexual power, for bruises inflicted by blows, skin diseases, as a soothing effect on the body, for wounds on the neck of bullocks and bleeding	Bark	Bark-powder paste is used, mucilage in the gum from the bark	[16]
Skin boils	Seed	Seed powder	[16]

**Table 2:** Chemical compounds isolated from *Litsea glutinosa*.

Phytochemicals	Part(s)	Reference(s)
Alkaloids		
Litsine A (**1**)	Root bark	[24]
Litseglutine A (**2**)	Leaves and twigs	[25]
Litseglutine B (**3**)	Leaves and twigs	[25]
Litsine B (**4**)	Root bark	[26]
Litsine C (**5**)	Root bark	[26]
Boldine (**6**)	Root bark	[24]
Laurolitsine (**7**)	Root bark	[24]
Glutinosine A (**8**)	Root bark	[27]
Morphinane (**9**)	-	[13]
Aporphine (**10**)		[13]
1-benzylisoquinoline (**11**)		[13]
Phenanthrene (**12**)		[13]
N-methylactinodaphnine (**13**)	Leaves	[28]
N-methyllaurotetanine (**14**)	Leaves	[28]
Isoboldine (**15**)	Leaves	[28]
Alcohols		
1-heptadecanol (**16**)	Bark	[29]
1-eicosanol (**17**)	Bark	[29]
Coclaurine (**18**)	Root bark	[30]
Dihydrobuddlenol (**19**)	Root bark	[30]
Ssioriside (**20**)	Root bark	[30]
Carbohydrates		
Xylose (**21**)	Leaves	[31]
Arabinose (**22**)	Leaves	[31]
Glycosides		
Litseasins A (**23**)	Root bark	[32]
Litseasins B (**24**)	Root bark	[32]
Litseasins C (**25**)	Root bark	[32]
Glutin (**26**)	Leaves and twig	[33]
(7R,8S)-3,3′,5-trimethoxy-4′,7-epoxy-8,5′-neolignan4,9,9-triol 9-β-D-xylopyranoside (**27**)	Root bark	[32]
(6S, 7E, 9R)-6, 9-dihydroxy-4, 7-megastigmadien-3-one-9-O-[α-L-arabinofuranosyl-(l→6)]-β-D-glucopyranoside (**28**)	Leaves and twig	[33]
Roseoside (**29**)	Leaves and twig	[33]
(7′R, 8′R)-3, 5′-dimethoxy-9, 9′-dihydroxy-4, 7′-epoxylignan 4′-β-D-glucopyranoside (**30**)	Leaves and twig	[33]
(7′R, 8′S)-dihydrodehydrodiconifenyl alcohol 9′-O-β-D-xylopyranoside (**31**)	Leaves and twig	[33]
Pinoresinol 3-O-β-D-glucopyranoside (**32**)	Leaves and twig	[33]
2′,5,7-trihydroxy-6-methoxyflavone 2′-O-β-D-glucopyranoside (**33**)	Leaves and twig	[33]
Schizandriside (**34**)	Root bark	[30]
Dendranthemoside B (**35**)	Root bark	[30]
Phenylethyl-β-D-glucopyranoside (**36**)	Root bark	[30]
N-cis-feruloyl tyramine (**37**)	Root bark	[30]
Esters		
cis-5,8,11,14,17-eicosapentaenoic acid methyl ester (**38**)	Bark	[29]
Spatozoate (**39**)	Bark	[29]
Glycerol 1,3-di-(9Z,12Z-octadecadienoate) 2-hexadecanoate (**40**)	Bark	[29]
Terpenes		
Phytol (**41**)	Leaf oil	[17]
Caryophyllene (**42**)	Leaf oil	[17]
Thujopsene (**43**)	Leaf oil	[17]
Myrcene (**44**)	Leaf oil	[17]
α-cubebene (**45**)	Fruit oil	[17]
β-ocimene (**46**)	Leaf oil	[17]
β-pinene (**47**)	Leaf oil	[17]
α-pinene (**48**)	Leaf oil	[17]
Caryophylleneoxide (**49**)	Leaf oil	[17]
Bicyclogermacrene (**50**)	Leaf oil	[17]
Ocimene (**51**)	Fruit oil	[17]
Flavonoids		
Flavones (**52**)	-	[13]
Flavonols (**53**)	-	[13]
Flavan-3-ols (**54**)	-	[13]
Chalcones (**55**)	-	[13]
Flavanonols (**56**)	-	[13]
Anthocyanidins (**57**)	-	[13]
Lactones		
Litsealactone C (**58**)	Bark	[11]
Litsealactone D (**59**)	Bark	[11]
Litsealactone G (**60**)	Bark	[11]
Eusmoside C (**61**)	Bark	[11]
(3R,4S,5S)-2-hexadecyl-3-hydroxy-4-methylbutanolide (**62**)	Bark	[11]
Steroids		
Stigmasterol (**63**)	Bark	[33]
Sitosterol (**64**)	Bark	[33]
β-sitosterol (**65**)	Bark	[29]
Epicatechin (**66**)	Bark	[15]
Vomifoliol (**67**)	Aerial parts	[34]
Daucosterol (**68**)	Aerial parts and bark	[29]
Pubinernoid B (**69**)	Aerial parts	[34]
Atroside (**70**)	Aerial parts	[34]
Miscellaneous constituents		
Lauric acid (**71**)	Fruit oil	[17]
3-octen-5-yne, 2,7-dimethyl (**72**)	Fruit oil	[17]
9,12-octadecadienoic acid (**73**)	Bark oil	[34]
Oleic acid (**74**)	Fruit oil	[17]
Benzyl alcohol-β-d-glucopyranoside (**75**)	Root bark	[30]
N-butyl-β-d-fructopyranoside (**76**)	Root bark	[30]
N-trans-sphingoyl tyramine (**77**)	Root bark	[30]

**Table 3:** Pharmacological activities of different parts of *Litsea glutinosa*.

Sources	Test Systems	Dose/Conc.	Results and Possible Mechanism	References
**Antioxidant activity**				
Methanolic extract of plant	Hydrogen peroxide scavenging activity, total antioxidant capacity, assay of nitric oxide scavenging activity and reducing-power test.	50–250 mg/mL	Exhibited antioxidant effect in a concentration-dependent manner.	[43]
Leaf extract	DPPH-free-radical scavenging assay, reducing-power assay, total phenolic content.	5–100 µg/mL	Antioxidant activity is dose-dependently increased.	[7]
**Anti-inflammatory activity**				
n-hexane, ethyl acetate, chloroform, and methanolic leaf extracts	Carrageenan-induced oedema test is carried out on *Swiss* albino mice.	250 and 500 mg/kg	The crude methanolic extract showed significant potential against carrageenan-induced paw edema, by inhibiting either cyclooxygenase and/or lypooxygenase enzyme.	[7]
**Anti-microbial activity**				
Methanolic bark-extract	*Staphylococcus aureus*, *Bacillus pumilus*, *Streptococcus pneumoniae*, *Escherichia coli*, *Bacillus subtilis*, *Lactobacillus arabinosus*, *Bacillus cereu*, *Sarcina lutea*, *Shigella dysenteriae*, *Shigella sonnei*, *Salmonella typhimurium*, *Vibrio cholera*, *Klebsiella pneumoniae*, *Escherichia coli.* using an agar-diffusion method	50–200 µg/mL	The bark extract is effectively used in diarrhea and dysentery by inhibiting both gram-positive and gram-negative bacteria.	[44]
Stem-bark and leaf-extracts	*S. aureus*, *B. stubtilis*, *E.coli*, *Pseudomonas aeruginosa*, *K. pneumoniae*, *S. typhi*, *Salmonella paratyphi and Proteus* sp. using agar-well diffusion method	40, 20, 10, 5 and 2.5 mg/mL	Potent antibacterial agent.	[45]
Ethanolic and water-soluble leaf- and bark-extracts	*E. coli*, *Enterobacter intermedium*, *Salmonella* sp., *S. aureus* and *Staphylococcus epidermis* using Kirby–Bauer disc-diffusion method	Ethanol extract (1000 μg/disc), Distilled-water extract (10 μg/disc)	Ethanolic leaf-extract showed maximum antibacterial activity against *E. coli* with a zone of inhibition of 30 mm.	[15]
Aqueous leaf-extract	*B. subtilis*, *Enterococcus faecalis*, *E. coli*, *K. pneumoniae*, *Micrococcus luteus*, *P. aeruginosa*, *Proteus vulgaris*, *S. aureus*, *S. pneumoniae*, *Aspergillus niger*, *Candida albicans and Saccharomyces cerevisiae.*	25, 50 and 100 mg/mL	Showed zone of inhibition of 50% more than the antibiotics investigated.	[8]
Ethanolic bark-extract	*S. aureus*, *B. cereus*, *P. aeruginosa*, *E.Coli*	125–1000 mg/mL	Showed effective antimicrobial effect in dose-dependent manner against the test microbes.	[46]
Methanolic leaf-extract	*Aedes aegypti* larvae	20, 40 and 60 g/L	At 60 g/L, the extract is considered to be most effective in larvicidal activity.	[47]
**Anticancer activity**				
New megastigmane diglycoside isolated from the plant.	Human cancer cell-lines myeloid leukemia HL-60, hepatocellular carcinoma SMMC-7721, lung cancer A-549, breast cancer MCF-7 and colon cancer SW480 cells.	(1 mg) in 1 mol L-1 HCl (2 mL)	Proved to be inactive (IC50 > 40 µM).	[39]
N-methylactinodaphnine, boldine, N-methyllaurotetanine, and isoboldine isolated from the ethanolic leaf-extract.	Cytotoxicity against HT29, SKMEL28, and primary human keratinocytes.	100 μg/mL	Exerted cytotoxic effect through inhibiting DNA topo-II.	[28]
Methanolic bark-extract	In vitro studies on Saos-2 cell	500 ng/mL, 10–400 μg/mL	Significantly downregulated the apoptotic and proliferative markers in Saos-2 osteocytes.	[48]
**Antipyretic activity**				
n-hexane, ethyl acetate, chloroform, and crude methanolic leaf-extracts	The subcutaneous injection of yeast suspension in *Swiss* albino mice.	500 mg/kg	Exerted notable reduction in yeast-provoked elevation of body temperature (32.78 ± 0.46 °C) through inhibition of prostaglandin synthetase within the hypothalamus.	[7]
**Anti-diabetic effect**				
Ethanol bark-extract	Male ob/ob mice.	50, 100 or 200 mg/kg	Ameliorated insulin resistance through alleviating obesity, hyperlipidemia and inflammation, and can be used as potent treatment of type 2 diabetes.	[49]
Glutinosine A isolated from the root bark.	HepG2 cells for glucose consumption assay.	10 μM	Exerted no activity in stimulating glucose-consumption.	[27]
Laurolitsine isolated from the plant.	ob/ob mice	2.0 mg/kg via the tail vein, 10.0 mg/kg by gavage	Demonstrated potent antihyperglycemic and antihyperlipidemic effect.	[50]
Litsine B and C isolated from the ethanolic root-bark extract.	Glucose-consumption assay on HepG2 cells.	1–20 μM	Litsine C b significant increasing glucose-consumption.	[26]
Litsine A isolated from the root bark.	Glucose-uptake assay on C2C12 myoblasts.	10 μM	Increased glucose uptake.	[24]
**Analgesic activity**				
n-hexane, ethyl acetate, chloroform, and crude-methanolic leaf extracts	Acetic-acid-induced writhing and hot-plate test in mice.	250 and 500 mg/kg	Displayed significant analgesic-activity.	[7]
Leaf extract	Abdominal-writhing and tail-flick methods, using mice.	100, 200 and 300 mg/kg	Provided significant analgesic activity by inhibiting prostaglandin synthetase, specifically endoperoxidase.	[45]
Methanolic extract of the plant	Acetic-acid-induced writhing model in Swiss albino mice.	250 and 500 mg/kg	Provided a dose-dependent increase in analgesic effect.	[43]
**Hepatoprotective effect**				
Methanolic extract of the plant	CCl_4-_ and paracetamol-induced hepatotoxicity in rats.	100–200 mg/kg	Provided potent hepatoprotective-effect with controlled biological parameters.	[51]
**Miscellaneous effects**				
Bark extract	Immobilization stress-induced male Wistar albino rats.	100, 300, and 500 mg/kg	Exhibited significant aphrodisiac and anti-infertility activity.	[46]
n-hexane, ethyl acetate, chloroform, and crude methanolic leaf extracts	Swiss albino mice.	1 mg/mL	A significant clot-disruption was observed.	[7]

## Data Availability

Data-sharing not applicable.

## Abbreviations

ABTS	2,2′-azino-bis (3-ethylbenzothiazoline-6-sulfonic acid)
BHT	Butylated hydroxytoluene
CCl_4_	Carbon tetrachloride
DPPH	1,1-diphenyl-2-picryl hydrazine
HL	Human leukemia
H_2_O_2_	Hydrogen peroxide
HCl	Hydrochloric acid
HT29	Human adenocarcinoma colorectal cell line
HepG2	Hepatoma G2
HPLC	High-performance liquid chromatography
LC/TOF-MS	Liquid chromatography/time-of-flight-mass spectrometry
MIC	Minimum inhibitory concentration
NMR	Nuclear magnetic resonance
SMMC-7721	Surface Marker and Micro Cell-7721
TLC	Thin-layer chromatography
